# High-resolution global peptide-protein docking using fragments-based PIPER-FlexPepDock

**DOI:** 10.1371/journal.pcbi.1005905

**Published:** 2017-12-27

**Authors:** Nawsad Alam, Oriel Goldstein, Bing Xia, Kathryn A. Porter, Dima Kozakov, Ora Schueler-Furman

**Affiliations:** 1 Department of Microbiology and Molecular Genetics, Institute for Medical Research Israel-Canada, Faculty of Medicine, The Hebrew University, Jerusalem, Israel; 2 School of Computer Sciences and Engineering, The Hebrew University, Jerusalem, Israel; 3 Department of Biomedical Engineering, Boston University, Boston, Massachusetts, United States of America; 4 Department of Applied Mathematics and Statistics, Stony Brook University, Stony Brook, New York, United States of America; 5 Laufer Center for Physical and Quantitative Biology, Stony Brook University, Stony Brook, New York, United States of America; 6 Institute for Advanced Computational Sciences, Stony Brook University, Stony Brook, New York, United States of America; Fox Chase Cancer Center, UNITED STATES

## Abstract

Peptide-protein interactions contribute a significant fraction of the protein-protein interactome. Accurate modeling of these interactions is challenging due to the vast conformational space associated with interactions of highly flexible peptides with large receptor surfaces. To address this challenge we developed a fragment based high-resolution peptide-protein docking protocol. By streamlining the Rosetta fragment picker for accurate peptide fragment ensemble generation, the PIPER docking algorithm for exhaustive fragment-receptor rigid-body docking and Rosetta FlexPepDock for flexible full-atom refinement of PIPER docked models, we successfully addressed the challenge of accurate and efficient global peptide-protein docking at high-resolution with remarkable accuracy, as validated on a small but representative set of peptide-protein complex structures well resolved by X-ray crystallography. Our approach opens up the way to high-resolution modeling of many more peptide-protein interactions and to the detailed study of peptide-protein association in general. PIPER-FlexPepDock is freely available to the academic community as a server at http://piperfpd.furmanlab.cs.huji.ac.il.

## Author summary

Peptide-protein interactions are crucial components of various important biological processes in living cells. High-resolution structural information of such interactions provides insight about the underlying biophysical principles governing the interactions, and a starting point for their targeted manipulations. Accurate docking algorithms can help fill the gap between the vast number of these interactions and the small number of experimentally solved structures. However, the accuracies of the existing protocols have been limited, in particular for *ab initio* docking when no information about the peptide beyond its sequence is available. Here we introduce PIPER-FlexPepDock, a fragment-based global docking protocol for high-resolution modeling of peptide-protein interactions. Integration of accurate and efficient representation of the peptide using fragment ensembles, their fast and exhaustive rigid-body docking, and their subsequent accurate flexible refinement, enables peptide-protein docking of remarkable accuracy. The validation on a representative benchmark set of crystallographically solved high-resolution peptide-protein complexes demonstrates significantly improved performance over all existing docking protocols. This opens up the way to the modeling of many more peptide-protein interactions, and to a more detailed study of peptide-protein association in general.

## Introduction

Proteins are the workhorses inside living cells, and interactions among them are critical for various important biological processes [1]. A significant fraction of these interactions (15–40%) [2] are peptide mediated, where a short stretch of residues from one partner contributes most to its binding to the other. Such short peptidic regions, also termed short linear interacting motifs (SLIMs) are often found embedded inside disordered regions of intrinsically disordered proteins (IDPs) [2, 3], or appear as flexible linkers connecting domains [4] and as flexible loops tethered to rigid segments [5].

The development of accurate structure based modeling tools is critical for atomic level understanding of peptide-protein interactions, to allow the manipulation of known interactions, to discover yet unknown peptide-protein interactions and networks, and to provide starting points for the design of novel peptides and related molecules to target specific systems of pharmacological interest [6]. A number of computational tools have been developed to assist the characterization of peptide-protein interactions, including the prediction of peptide binding sites [7–9], refinement of coarse peptide-protein models [10], folding and docking on a known binding site [11] and most challenging of all, global peptide-protein docking with no prior information about the peptide structure and the binding site [12–17]. The challenges associated with the global docking of flexible peptides have been addressed in different ways, by reducing the conformational space to be sampled both for the internal degrees of freedom of the peptide as well as its rigid-body orientations on the receptor surface. For peptide docking within the HADDOCK docking framework [12], the peptide backbone is represented by idealized conformation(s), such as alpha helix, beta strand and polyproline-II, followed by rigid-body, semi-flexible and fully-flexible docking with explicit solvation [18]. The pepATTRACT protocol [13, 19] uses the same approach to represent the peptide, followed by coarse-grained rigid-body docking and flexible full-atom refinement. The AnchorDock protocol uses molecular dynamics simulations to generate a set of plausible peptide conformations, which are then docked using anchor-driven simulated annealing molecular dynamics around predicted anchoring spots on the receptor [14]. The CABS-dock protocol uses randomly generated peptide conformations based on either predicted or known secondary structure, randomly orients these peptides over the receptor surface, and refines them using replica exchange Monte Carlo dynamics [15]. The MDockPep protocol [16] uses peptide sequence similarity to extract fragments from high resolution protein structures, which are further refined using MODELLER [20] to generate plausible peptide conformations, and then docked onto the receptor using rigid-body docking and flexible docking with AutoDock Vina [21]. The recently published IDP-LZerD protocol models the binding of long disordered segments to structured proteins using the Rosetta fragment picker protocol [22] to generate fragments of 9-residue overlapping windows followed by LZerD [23] rigid-body docking and molecular dynamics refinement [17]. Finally, we have recently advanced a novel, global motif-based peptide fragment docking approach, PeptiDock [24], in which peptide binding motif information rather than secondary structure propensity is used to extract fragments from the Protein Data Bank (PDB [25]), which are then docked to the receptor using PIPER rigid body docking [26], followed by minimization using CHARMM [27].

Notwithstanding these significant recent advances in global peptide docking, present approaches are still limited in their modeling quality and general applicability, and there is ample room for improvements that would enable the detailed high-resolution study of more peptide-protein interactions with higher accuracy. Here we describe PIPER-FlexPepDock, a successful effort toward the development of such a robust, highly accurate, global peptide-protein docking protocol. By integrating accurate peptide fragment ensemble generation using the Rosetta fragment picker [22], fast and exhaustive fragment-receptor rigid-body docking using PIPER docking [28], and flexible full-atom refinement of coarse PIPER models using Rosetta FlexPepDock [10], we were able to sample both the peptide backbone conformational states, as well as the landscape of the peptide-receptor interactions efficiently and with much higher accuracy than current protocols: on a representative non-redundant dataset of peptide-protein complexes well resolved by X-ray crystallography (Table 1 below), PIPER-FlexPepDock generates for about half models within 2.5 Å ligand RMSD (2.0 Å, when restricted to motif regions where available), more than twice as many as for existing peptide docking protocols such as pepATTRACT [13] (among the 10 top-ranked predictions; Table 2 below).

**Table 1:** Benchmark of peptide-protein complexes used in this study (non-redundant set; see S1C Table for full set). PDB ids of the initial calibration set are highlighted in bold.

PDB ID	Peptide sequence^a^/ secondary structure	Fragment similarity^b^	PIPER^c^	PIPER-FPD *(motif)*					PIPER-FPD *(full peptide)*				
Complex/Free Receptor			L^d^	L		I^e^		Fnat	L	I		Fnat	
**Known binding motif (n = 12)**													
**1CZY:CE** **1CA4:A**	PQQATDD CEECCCC	2.2(2.5)	17.6	**1.6**	0.6		0.86		**2.4**		0.9		0.76
1EG4:AP 1EG3:A	NMTPYRSPPPYVP TTTTTTCCCCCCC	0.6(3.0)	21.6	12.9	4.1		0.19		29.3		11.5		0.00
1ELW:AC 1A17:A	GPTIEEVD CCCCCCCC	1.0(2.4)	3.2	**0.8**	2.5		0.75		2.6		3.0		0.71
**1JD5:AB** **1JD4:A**	AIAYFIPD CEEEETCC	0.7(2.3)	2.8	**1.2**	0.5		0.88		8.2		2.9		0.19
**1JWG:BD** **1JWF:A**	DEDLLHI CCCCCCC	2.8(2.9)	3.4	**2.2**	0.8		0.90		**2.2**		0.8		0.90
**1MFG:AB** **2H3L:A**	EYLGLDVPV CCCCCCEEC	1.5(2.7)	3.1	**1.8**	0.8		0.73		8.7		3.1		0.29
1NTV:AB 1P3R:B	NFDNPVYRKT CEETTTTCCC	2.7(3.3)	5.6	5.2	1.7		0.43		3.8^f^		1.5		0.52
1RXZ:AB 1RWZ:A	KSTQATLERWF CEEECTTTTTC	2.4(4.0)	7.4	5.0	1.9		0.31		3.2		1.7		0.39
1SSH:AB 1OOT:A	GPPPAMPARPT CCCCCCCCCCC	1.1(2.3)	2.6	7.6	2.9		0.56		**1.9**^f^		1.1		0.87
1X2R:AB 1X2J:A	LDEETGEFL CTTTTTCCC	0.2(0.5)	**1.1**	**1.3**	0.5		0.74		**1.7**		0.6		0.72
**2A3I:AB** **2AA2:A**	QQKSLLQQLLTE CCCCHHHHHHHC	0.3(3.5)	**1.6**	**1.0**	0.4		0.93		4.8		2.1		0.72
**2CCH:DF** **1H1R:B**	HTLKGRRLVFDN TTTTCCCCCCCC	1.8(4.6)	**3.4**	**1.0**	0.4		0.91		3.9		1.6		0.67
**No known binding motif (n = 15)**													
1AWR:CI 2ALF:A	HAGPIA CCCCCC	1.6	6.5						**1.3**		0.5		0.97
**1ER8:EI** **4PAE:A**	HPFHLLVY CCCBCCBC	1.9	4.1						**1.2**		0.8		0.80
1LVM:AE 1LVB:B	ENLYFQ CCEEEC	1.9	**2.3**						**1.4**		0.6		0.91
1NVR:AB 2QHN:A	ASVSA CEEEC	2.0	5.5						7.1		2.2		0.56
1NX1:AC 1ALV:A	DAIDALSSDFT HHHHHHHHHCC	1.8	1.9						**1.3**		0.9		0.80
1OU8:BD 1OU9:A	GAANDENY CCCCCCCC	3.1	6.1						6.4		2.4		0.39
1U00:AP 2V7Y:A	ELPPVKIHC CCCCEECCC	2.3	6.0						**2.1**		1.7		0.71
2B9H:AC 2B9F:A	RRNLKGLNLNLH CCTTTTCCCCCC	3.2	16.4						15.8		6.3		0.18
2C3I:BA 2J2I:B	KRRRHPSG CCCCCCCC	2.5	3.5						8.7		2.5		0.26
**2DS8:BP** **2DS7:A**	ALRVVK CCEECC	1.4	4.7						**1.2**		0.6		0.86
2FMF:AB 1JBE:A	QDQVDDLLDSLGF HHHHHHHHHHHCC	1.1	5.2						**1.3**		0.7		1.00
2H9M:CD 2H14:A	ARTKQ TTTTC	2.2	4.7						4.0		1.1		0.37
**2HPL:AB** **2HPJ:A**	DDLYG CCCCC	1.6	3.0						**1.4**		0.5		0.91
2O02:AP 2BQ0:A	GLLDALDLAS THHHHHCCCC	2.4	2.8						3.4		1.2		0.74
3D1E:AP 3D1G:A	GQLGLF CBCCCC	2.5	4.1						6.7		2.0		0.51

^*a*^ Motif (as defined by the ELM database [29]) is underlined (motif details are provided in the S1B Table).

^*b*^ Similarity between fragments and bound peptide conformation: Median backbone RMSD (Å) (in parentheses: results for full peptide).

^*c*^ Results for PIPER simulations are given for the motif / full peptide for known/unknown motifs, respectively. The models are selected as in PeptiDock [24] (without the minimization step).

^*d*,*e*^ Modeling accuracy: L–Ligand RMSD (models within 2.5 Å are highlighted in bold)^*c*^; I–Interface RMSD ^*d*^. Defined as in the CAPRI experiment [34, 35].

^*f*^ Complexes for which docking of the full peptide provides better models than docking of the motif only.

**Table 2:** Summary of performance of PIPER-FlexPepDock, and comparison to other peptide docking protocols. Results are shown for PIPER-FlexPepDock runs on unbound receptor structures, including receptor minimization.

Cutoff L^a^	PIPER-FlexPepDock		pepATTRACT [13]	HADDOCK [12]	CABSDOCK [15]	MDockPep [16]
	motif	full				
non-redundant set (n = 27)						
1.5	12 (44%)	7 (26%)	2 (7%)	0	0	0
2	14 (52%)	9 (33%)	3 (11%)	1 (5%)	0	0
2.5	16 (59%)	12 (44%)	4 (15%)	2 (9%)	1 (4%)	0
3	16 (59%)	13 (48%)	7 (26%)	5 (23%)	3 (11%)	1 (4%)
calibration set (n = 9)						
1.5	6 (67%)	3 (33%)	1 (11%)	0	0	0
2	8 (89%)	3 (33%)	2 (22%)	0	0	0
2.5	9 (100%)	5 (56%)	2 (22%)	1 (11%)	0	0
3	9 (100%)	5 (56%)	4 (44%)	1 (11%)	1 (11%)	0
redundant set (n = 42)^b^						
1.5	20 (48%)	10 (24%)	4 (10%)	1 (3%)	1 (2%)	0
2	26 (62%)	17 (40%)	7 (17%)	3 (9%)	2 (5%)	1 (2%)
2.5	29 (69%)	22 (52%)	8 (19%)	4 (12%)	5 (12%)	2 (5%)
3	29 (69%)	25 (60%)	13 (31%)	11 (32%)	7 (17%)	4 (10%)

^*a*^ number of models within the mentioned ligand RMSD value (Å)

^*b*^ n = 34 for HADDOCK results

Our results highlight the relevance of representing the peptide as a set of fragments that can be exhaustively docked as rigid bodies onto the receptor structure and subsequently refined using an accurate refinement protocol. They reinforce the underlying biophysical model of a conformer ensemble of the free peptide that already samples the bound conformation (at least in the encounter-complex, protein-like environment) and involves only limited induced fit, not unlike the classical association between preformed protein domains. As a result, PIPER-FlexPepDock brings into reach the study and targeted manipulation of a range of additional peptide-mediated interactions not accessible before due to limitations in sampling and/or accuracy.

## Results

### Overview of the PIPER-FlexPepDock protocol (Fig 1)

**Fig 1:**
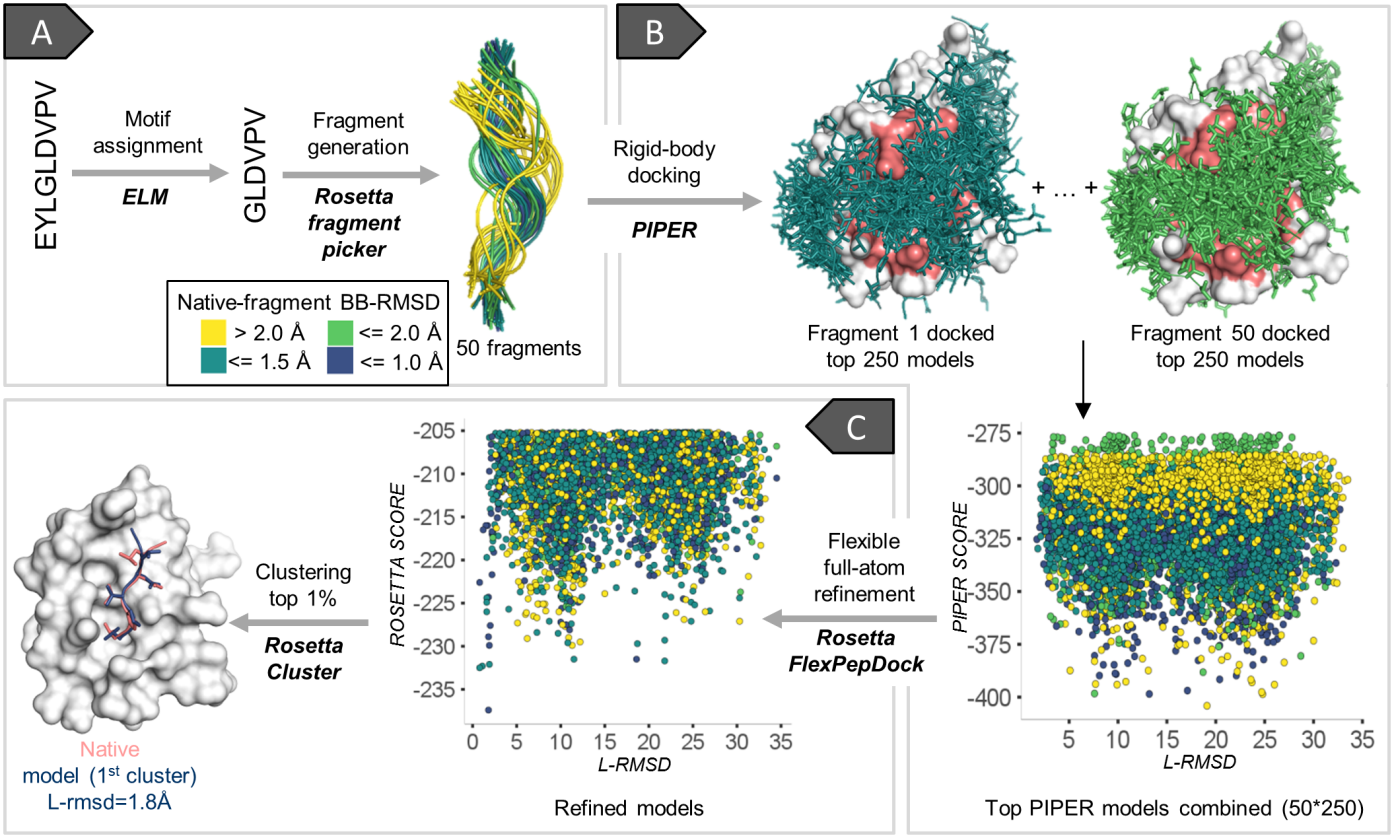
Overview of the PIPER-FlexPepDock peptide docking protocol. Example shown: PDZ domain-peptide interaction [PDB IDs of receptor structure 1MFG (bound) and 2H3L (free)]. For a given receptor structure and peptide sequence, the divide and conquer strategy involves first the description of the peptide as an ensemble of fragments **(A)**, their fast and exhaustive rigid body docking (using PIPER) onto the whole receptor (binding site region is shaded salmon) **(B)**, and subsequent high-resolution refinement (using Rosetta FlexPepDock; the top 5000 models are included in the plot) **(C),** followed by clustering and selection of top ranking representatives. Fragments are colored according to their similarity to the native bound peptide conformation. L-RMSD: Ligand root mean square deviation from crystal structure; see text for more details.

#### Step A | Generation of fragment set to represent the peptide conformer ensemble

In a previous study we have shown that the bound peptide conformation can be well represented by extraction of short fragments from the PDB based on information of known binding sequence motifs [24]. Here we have generalized this approach beyond motifs, using fragment libraries selected by the Rosetta fragment picker protocol [22] based on sequence and secondary structure similarity (see Methods). The coordinates of the top 50 mapped fragments are extracted from the PDB, including both backbone and side-chain atoms, and non-identical residues in the extracted fragments are mutated to the desired sequence. This set of fragments adequately represents the peptide conformational ensemble, sampling also its receptor bound conformation (see below). The peptide may be trimmed in cases where information is available about the range of the binding segment (from motif databases such as the Eukaryotic Linear Motif (ELM) resource [29, 30], literature, or experiments such as alanine scanning), since fragments generated for shorter peptide sequences are usually better representative than longer fragments (as, e.g., for loop modeling [31]), and fraying ends beyond the motif may contribute less to determine critical binding details.

#### Step B | Fragment rigid-body docking using PIPER

Each of the fragments is docked onto the receptor structure using PIPER, an exhaustive Fast Fourier Transform (FFT)-based rigid body docking algorithm [28], as implemented previously for PeptiDock [24] (see Methods), and top ranking fragment orientations from each docking run are collected and combined together. These models are of low resolution as no flexibility is included in the PIPER algorithm, and therefore ranked using a soft potential that allows a certain degree of steric clashes to overcome the limitations of rigid-body only docking.

#### Step C | FlexPepDock refinement of PIPER models and selection of final models

Each of the PIPER models is refined by a single fully flexible refinement run using the Rosetta FlexPepDock Refinement algorithm [10] (see Methods). The top ranking refined models are clustered (as in Gray *et al*. [32]), clusters are ranked based on the reweighted score of the best scoring model in each cluster (as in Raveh *et al*. [11]), and the top 10 ranked cluster representatives are selected as prediction (following the CAPRI scheme that accepts 10 models [33]).

### Initial calibration of the PIPER-FlexPepDock on a small set of protein-peptide complexes

Motivated by our recent advance in global peptide docking using a motif-focused approach [24] we ventured into the development of a more generalized protocol. We initially calibrated our docking approach on a small but representative set of nine peptide–protein complexes (highlighted in bold in Table 1; see also S1A Table). We trimmed the peptide based on the motif defined in ELM, where available. For all complexes high modeling accuracy was achieved for this new global docking approach (within ≤2.5Å Ligand RMSD models among the top 10 ranking clusters; Table 1). For the full length peptides modeling near-native models were obtained for 5/9 cases, highlighting the benefits for motif (or shorter peptide sequence) focused modeling, due to better fragment quality compared to the corresponding full-length peptides (Table 1). Encouraged by these initial results, we proceeded to the validation of our protocol on a larger representative set of peptide-protein complexes (Table 1 and S1B Table).

### Assessment of peptide docking performance

We assessed the performance of PIPER-FlexPepDock on a larger, non-redundant set consisting of 27 complexes (compiled from the 42 complexes used in previous studies, but non-redundant at the domain level, as defined by CATH [36]; see Methods), among them 12 with reported binding motif. The benchmark is summarized in Table 1 (S1C Table provides results for the redundant set of 42 complexes used in previous studies, as well as additional details, including performance of other approaches for comparison).

#### Representing the peptide conformational states using fragments

Fragments derived from solved protein structures contain valuable information about the local structural context that can be used to efficiently reduce sampling space for various modeling applications, including e.g. *ab initio* protein folding [37] and loop building [31, 38, 39]. In our protocol we use the Rosetta fragment picker protocol [22] to generate fragments consistent with both the peptide sequence and the (predicted) secondary structure (See Methods).

**Fig 2:**
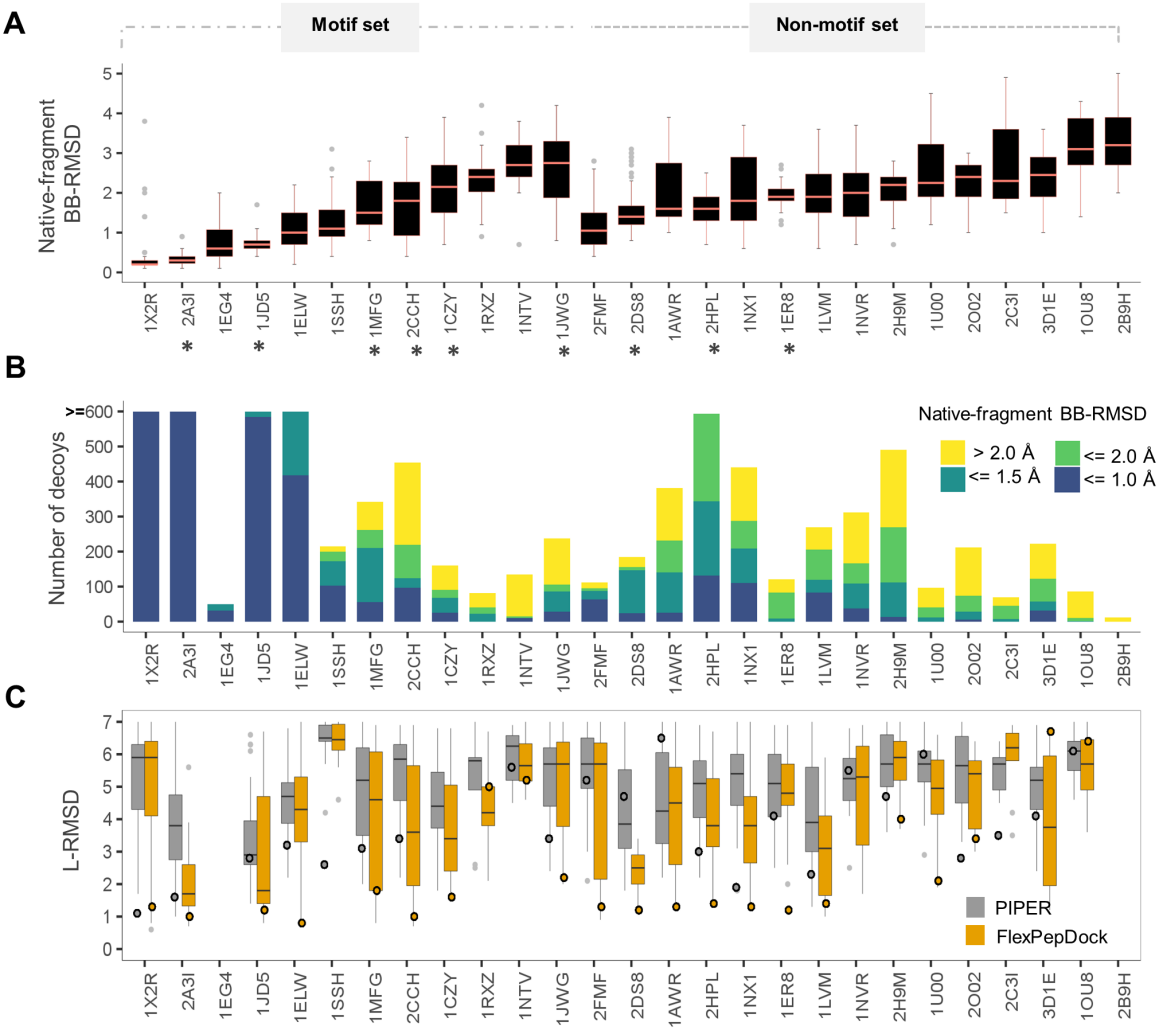
Assessment of performance of the different steps of PIPER-FlexPepDock. **(A)** Fragment quality: distribution of fragment backbone RMSDs relative to the native bound peptide conformation (defined as fragment quality). PDBs with and without motif information are grouped separately. The initial calibration set is marked with asterisks (*). **(B)** PIPER rigid body docking: distribution of the number of models within 5Å ligand (L)-RMSD from the native, colored according to fragment quality. **(C)** Improvement after FlexPepDock refinement: distribution of the L-RMSDs of the top 1% FlexPepDock refined models (in orange) and corresponding PIPER models (in gray). Shown are the results of runs starting from the unbound receptor structure and including receptor minimizations (see also Fig 3). The circles represent the L-RMSD values of the best model among the top 10 ranking clusters. The Y-axis has been trimmed to 7Å. Note that for the PIPER runs, circles represent the top-ranked model of a PIPER run (including density clustering, as described in Methods and Porter *et al*. [24]), while the distributions represent the subset of models that served as starting structures for the models selected after FlexPepDock refinement. The former allows the comparison of the final results from a PIPER run to a corresponding PIPER-FlexPepDock run, while the latter shows improvement due to FlexPepDock refinement for the finally selected models.

How accurately do the fragments represent the peptide conformational states? Most importantly, how similar are peptide conformations to the one adapted when bound to their receptor? A significant representation of similar fragments could guarantee that, when docked with high density in the binding site using an exhaustive but accurate rigid-body docking algorithm, they could efficiently be refined to high resolution using an accurate refinement algorithm such as Rosetta FlexPepDock. To assess the quality of the fragments (*i*.*e*., their coverage of the bound conformation) we analyzed the distribution of backbone RMSDs of the fragments relative to the bound peptide conformation. Reassuringly, the fragment pool generated using the Rosetta fragment picker protocol represents in most cases the bound like peptide conformation with high accuracy in our benchmark of 27 peptide-protein complexes (Fig 2A: median backbone RMSD within 2.0 Å for 15 out of the 27 cases, with average backbone RMSD of the best ten fragments within 1.0 and 1.5 Å for 14 and 21 cases, respectively). The best accuracy is achieved for helical peptide motifs (e.g., the helical nuclear receptor box motif in 2A3I [40]; for helical peptides with coiled terminus segments such as 2FMF [41] and 1NX1 [42] the median backbone RMSD is slightly higher). Even for the remaining peptides the fragment ensemble is often composed of a significant portion of bound like representatives. The worst representation of bound-like peptides is obtained for few longer coil peptides, such as 2B9H [43], which defines the limitation of the fragment picker protocol for longer sequences. In such cases, trimming the peptide might improve the quality significantly.

We previously showed that extracting fragments based on sequence motif information allows identification of bound peptide conformations that reflect the structural pattern of these motifs [24]. We demonstrate here that representative fragments are not restricted to peptides with known motifs. In fact, a comparison to the fragments extracted based on sequence motif (for the dataset analyzed in the PeptiDock study, using the motif definition therein [24]) shows that the fragment picker approach produces overall ensembles that contain structures more similar to the bound peptide conformation (see S2 Table).

#### Rigid-body docking: Fragment quality and PIPER performance

The fact that the fragment ensembles include bound-like conformations justifies proceeding to the next step, namely their docking onto the receptor. The PIPER rigid-body docking protocol allows fast and exhaustive sampling to provide coarse models of fragment-receptor interactions, of which the top-scoring can be followed up by subsequent refinement to allow for conformational changes upon binding. The effective range for successful refinement using the FlexPepDock protocol was previously found to be within up to 5Å in terms of Cartesian RMSD, and up to 50 degrees in terms of Φ–Ψ RMSD (distance of fragment from the bound peptide conformation in Φ–Ψ dihedral space) [10]. It is thus important for the PIPER docking stage to identify a large pool of fragments that are densely docked in close proximity (within effective Cartesian RMSD range) of the native peptide binding mode, involving docked fragments that are similar to the native peptide bound conformation (within effective phi-psi RMSD range). Indeed, analysis of the top ranking PIPER models shows presence of good quality fragments at the binding site (in fact, most complexes include <1.0Å bb RMSD fragments; Fig 2B).

#### Improvement of PIPER models by FlexPepDock refinement

**Fig 3:**
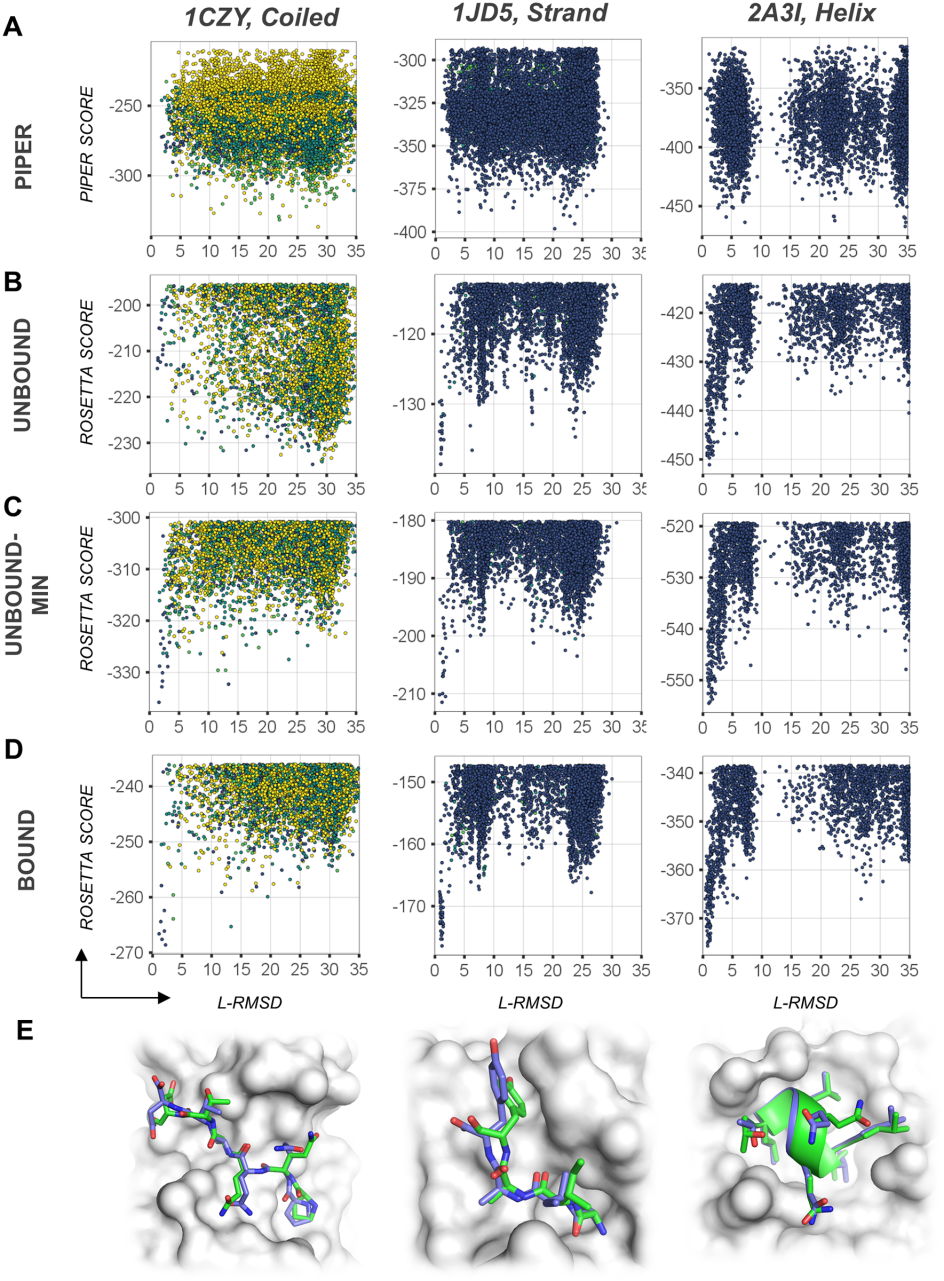
Examples of global peptide docking energy landscapes. **Left:** PDB id 1CZY (coiled peptide); **Center**: 1JD5 (extended peptide); **Right:** 2A3I (helical peptide). **(A)** Energy landscape as sampled in the first docking step of the protocol by PIPER rigid body docking of peptide fragments onto the *unbound* receptor structure. **(B-D)** Energy landscapes for the PIPER-FPD scheme, starting from the unbound receptor structure **(B)**, the unbound receptor structure including receptor flexibility **(C)**, and the corresponding bound receptor for comparison **(D)**. Models are colored according to fragment quality, as in previous Figures. **(E)** Comparison of the modeled to the native structure (shown in blue and green, respectively).

The FFT algorithmic implementation of rigid-body sampling in PIPER makes exhaustive orientation search possible with significant computational efficiency, but is defined on a grid. Consequently, the scoring function can successfully isolate the best few hundreds from the vast pool of billions of positions of the peptide fragment relative to the receptor, but not discriminate the top rigid-body docked models further (Fig 3A & S1 Fig). In turn, the Rosetta scoring function used in the FlexPepDock Refinement protocol (currently Talaris 2014 [44]) is highly accurate, but this flexible docking protocol lacks the ability for fast and exhaustive sampling. Thus, to address the problem of exhaustive sampling with high accuracy, we combine the fast and exhaustive rigid-body sampling of PIPER with accurate flexible refinement by FlexPepDock of the top ranking few hundred best models. Indeed, the FlexPepDock refinement stage significantly improves the model quality, as well as better model ranking (See Figs 2C and 3C and S1 Fig). This includes the identification of a near-native funnel missed before (e.g. 1CZY in Fig 3 –compare A to C), or significant enhancement of a near-native funnel (e.g. 1JD5 and 2A3I). More examples can be found in S1 Fig.

**Fig 4:**
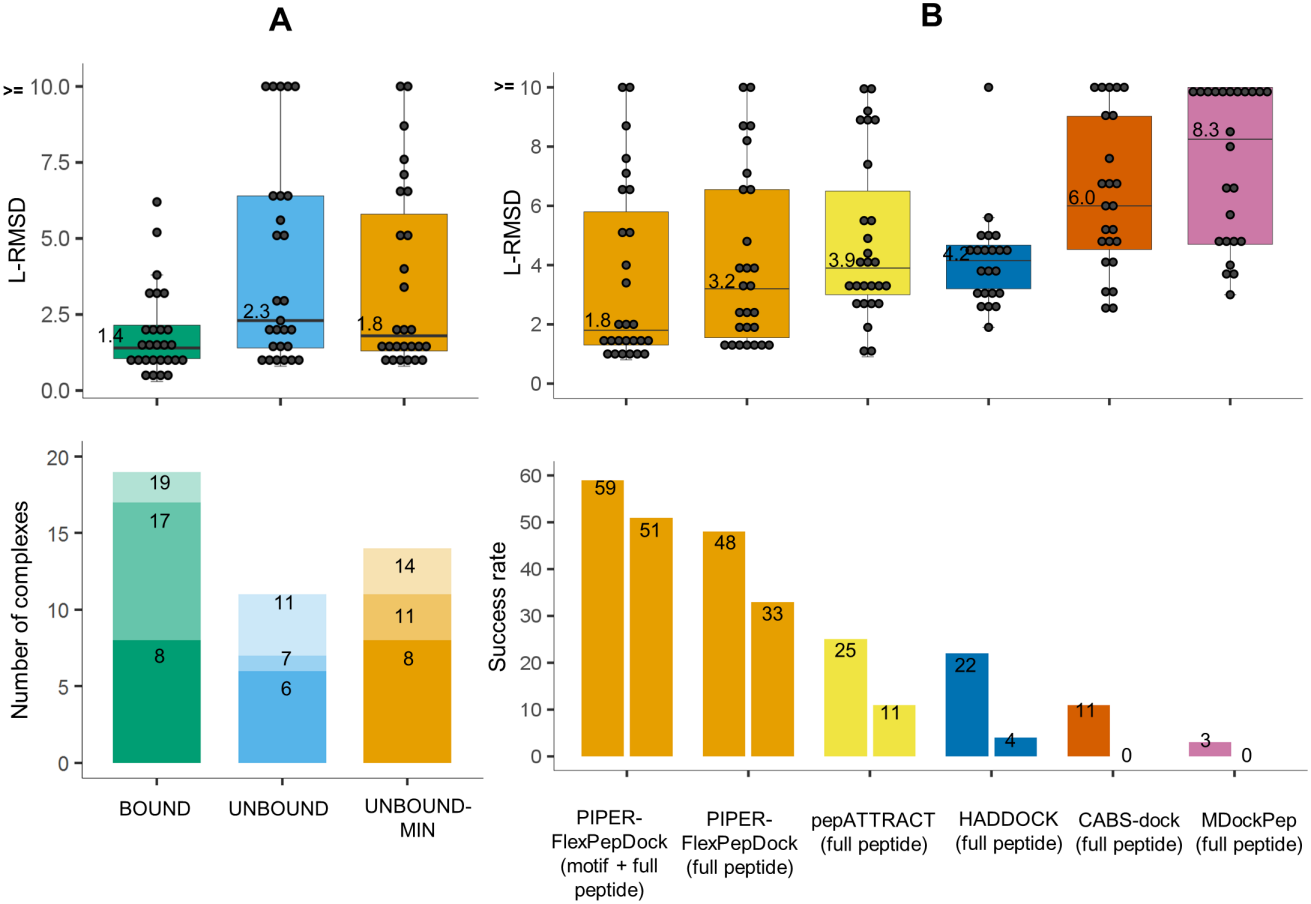
PIPER-FlexPepDock peptide docking performance. **(A)** Overall performance on a non-redundant set of 27 peptide-protein complexes. **Top:** Distribution of best model L-RMSDs (among top 10 ranking clusters) for runs using the bound (BOUND) and free (UNBOUND & UNBOUND-MIN) receptor structures, the latter including also receptor flexibility in the final refinement step (only the motif region was modeled for the 12 complexes with known motif). Shown are both the L-RMSD values for each protein-peptide complex (grey circles, rounded values for improved visibility are provided), as well as the distribution (quartiles and medians, with median values printed alongside). **Bottom:** Distribution of the ranks of the first near-native cluster (defined as L-RMSD < = 2.0Å), shown using different shades (for corresponding results among the top1, top3 and top10 ranked predictions). **(B)** Comparison to performance by other algorithms. Top: Box plots of best L-RMSDs among top 10 ranking clusters, including results for the motif part where the motif is known (as in A), as well as for the full peptide, for comparison. Bottom: Performance is shown for different cutoffs (3.0Å and 2.0Å L-RMSD in left and right boxes, respectively) (See S1B Table for more details).

We performed three runs to assess protocol performance (Summarized in Fig 4A and S1B Table; specific examples are shown in Fig 3): First, we applied the protocol to bound receptor structures. For these runs a near-native peptide conformation (L-RMSD < = 2.0Å, see Methods section) was found among the top 10 ranked clusters for 19 out of 27 complexes (success rate = 70%, Fig 3D). We then proceeded to the real-world scenario, in which the free receptor structure was provided as starting point (unbound run), leading to worse performance, as expected (10 complexes successfully modeled—success rate = 37%, Fig 3B). Importantly however, when including also receptor flexibility during the refinement stage (unbound-min run), these results improved, in particular if 10 best models are considered (14/27 complexes successfully modeled—success rate = 52%, Fig 3C).

### Comparison with other global docking protocols

We compared the results of PIPER-FlexPepDock (unbound-min run) with other existing global peptide-protein docking protocols such as HADDOCK [12], pepATTRACT [13], CABS-dock [15], and MDockPep [16] on our non-redundant set of 27 complexes, as well as on the set of 42 complexes used by these protocols in previous studies [34 complexes were compared with HADDOCK as other 8 cases were not included in their unbound run set). Since full length peptides were modeled using the other protocols, we modeled full length peptides for the motif set cases for valid comparison. The success rate for generating near-native models (*i*.*e*., L-RMSD within 2.0Å, or 3.0Å) was significantly better for PIPER-FlexPepDock than any other protocol, even for models of the full peptides (see Fig 4B and Table 2).

### The PIPER-FlexPepDock server for the high-resolution modeling of peptide-protein interactions

**Fig 5:**
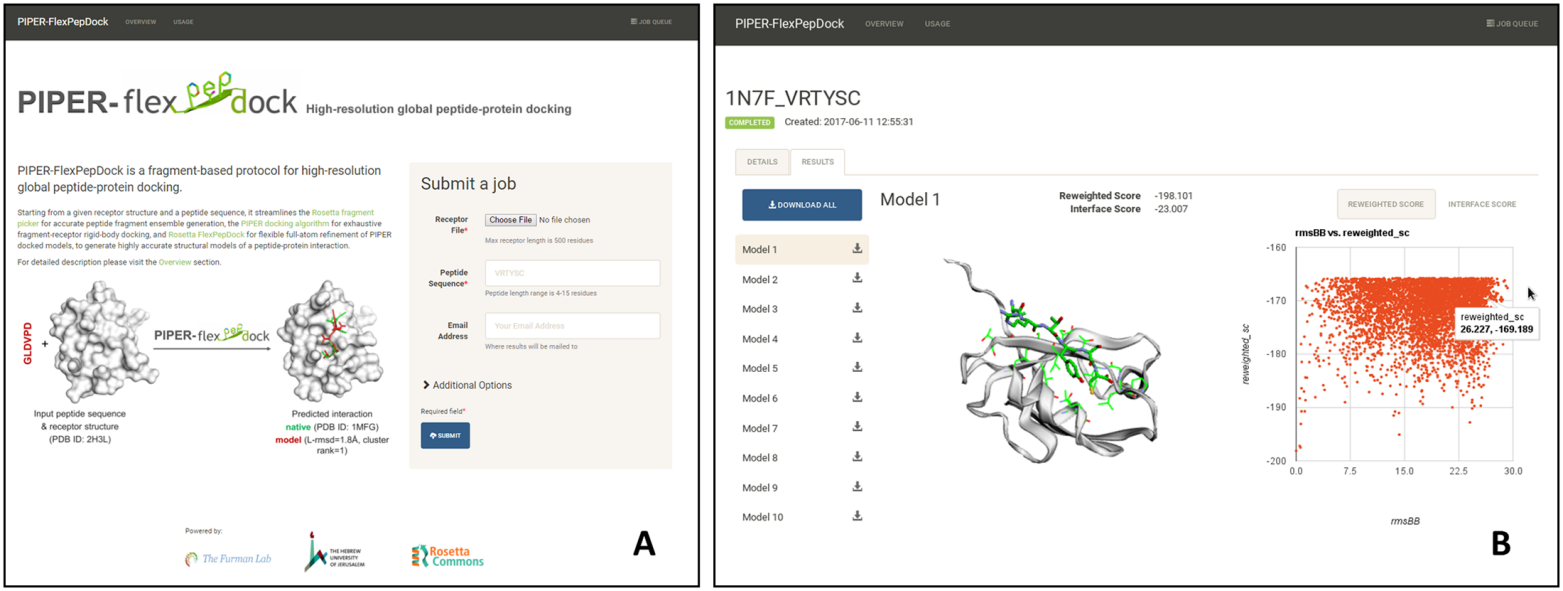
The PIPER-FlexPepDock server. **(A)** Job submission page: the required input includes the structure of the receptor and the sequence of the peptide; advanced options are accessible via a button. The tabs at the top provide links to detailed descriptions of the server, as well as to the Queue (upper right). **(B)** Results of an example peptide docking run: The liprin C-terminal peptide sequence VRTYSC docked onto the PDZ domain of GRIP1 (free receptor PDB id 1N7E). The top10 ranking models can be downloaded, and links to the individual models are provided to the left for inspection using an interactive viewer. In this case, Model 1 is an accurate prediction (L-RMSD = 1.0Å from solved structure PDB id 1N7F). On the right side a scatter plot shows the sampled energy landscape (relative to the lowest energy structure of the simulation).

In order to maximize the impact of our new protocol for global peptide-protein docking and to make it accessible to the modeling of many new peptide-protein complexes, we have set up a user-friendly server open to the scientific community (Fig 5). All that is needed is a structure of the receptor and a sequence of the peptide, but additional information about peptide secondary structure can also be included to narrow the search. The top-ranking resulting models can be downloaded, or inspected by an interactive viewer using the 3Dmol.js libraries [45].

## Discussion

### A new approach for global peptide docking with excellent performance

With the presentation of our new PIPER-FlexPepDock algorithm, we have demonstrated that combining fast and exhaustive rigid-body docking (using the FFT-based PIPER docking algorithm) of a representative peptide conformer ensemble (approximated by fragments extracted from solved structures, based on local similarity of sequence and secondary structure), with high-resolution refinement (using Rosetta FlexPepDock) is a successful approach for the generation of models of peptide-receptor structures of remarkable accuracy–significantly better than any other current protocol—starting from the sequence of the peptide and the structure of the receptor. The performance on a representative benchmark of solved peptide-protein complex structures demonstrates both accuracy and robustness of our modeling approach, and opens up the way of modeling many more peptide-protein interactions at much higher resolution and accuracy than any existing global peptide-protein global docking protocol.

### Receptor-bound peptide conformations are adequately represented by fragments extracted from protein monomer structures

This study demonstrates that fragments derived from solved protein structures, based on secondary structure and sequence similarity (rather than on sequence binding motifs which are not always available) represent the peptide conformational states with high accuracy, in particular the bound state. Interestingly, it is this same observation regarding the representation of local conformational preference that provided originally the platform for the breakthrough of Rosetta *ab initio* protein structure prediction [46]. This indicates that while isolated peptides in solution rarely show significant conformational preferences [47], in the encounter complex regime in vicinity of other proteins, their conformational freedom seems to be restricted significantly (similar to local peptide regions within a full protein) and can be represented by fragment libraries, in concordance with previous reports that show similar arrangements of fragments within monomers and peptide-protein interactions [48].

### Effective sampling of the energy landscape

The simplified scoring function and exhaustive sampling with PIPER allows uniform sampling of the fragments onto the receptor on a smoothened energy landscape. The top scoring PIPER models represent the dense sampling into wider energy basins. Though the ranking of models might lack the accuracy at this stage, the following refinement stage performs local sampling to efficiently locate the minimum. Interestingly, this approach is much more effective than the local refinement starting from one representative model: only one FlexPepDock optimization run is necessary starting from each PIPER model, compared to several hundred to thousand runs starting from a representative (defined, e.g. from a PIPER run as implemented in the PeptiDock peptide motif docking algorithm [24]). This is most probably due to the fact that these starting coarse models are trapped in many distinct states, each near a distinct local minimum, simplifying sampling during optimization.

### Mapping encounter complexes and more

The peptide-receptor binding energy landscape can provide a broader understanding of the binding mechanism itself. The exhaustive sampling with accurate refinement provides a high-resolution map of the energy landscape and helps us understand the energetic of the encounter between the peptide and the receptor. In a previous study, we were able to demonstrate that experimentally observed encounter complexes are well reproduced from a global protein docking energy landscape [49], and we anticipate that the corresponding peptide-protein docking energy landscape will provide similar information.

### Upcoming challenges

The approach described in this study improves significantly both accuracy as well as scope of peptide docking, at least as suggested by its performance on the widely accepted PeptiDB peptide docking benchmark [12, 13, 15, 16]. At the same time, it also highlights the bottlenecks to be overcome for its broader generalization: (1) ***Accurate modeling of peptide conformational ensemble*:** Even though the fragments generated using the Rosetta fragment picker protocol sample in general the bound peptide conformation well, challenges remain in the modeling of longer peptides, as well of as peptides with unusual conformations (Fig 2A). This is attributed to the lack of a large pool of longer representative fragments with similar sequences in solved structures. The rigid body PIPER docking step does not include any flexibility, and therefore accurate fragments are very important for efficient further refinement by FlexPepDock to near-native model quality. This challenge could be overcome by incorporating a peptide-folding algorithm as first step for fragment generation, assuming that bound-like conformations would indeed be sampled. (2) ***Modeling significant receptor backbone flexibility*:** While for many peptide-protein interactions the receptor is already pre-organized and the binding of the peptide does not induce considerable movement [50], binding may involve significant structural rearrangement of the receptor (e.g., in the binding of Slam tail peptide to the SH2 domain of the XLP protein SAP, PDB id 1D4T[51]). To model such challenging cases, improved modeling of receptor flexibility is mandatory (using e.g. backrub moves [52] and other advanced comparative modeling approaches [53]). (3) ***Improved ranking of alternative models*:** Inspection of failures highlights that despite low quality, many of the failed simulations model the peptide into the correct binding pocket, and identify the binding hotspot regions, similar to our observation in CAPRI community-wide performance [54]. However, the details are not correct, often pointing the wrong peptide residue side chain into a given binding pocket. Such ranking problems might be removed with the advance towards better scoring functions. (4) ***Extension to flexible interactions***: Last but not least, this approach might be restricted to peptide-mediated interactions in which the bound peptide adopts one, defined conformation, since it has been calibrated on well-resolved crystal structures of peptide-protein complexes. Many biologically relevant interactions remain more flexible, and are therefore studied using e.g. NMR experiments. The next challenge will be to extend this approach to the study of such interactions.

To summarize, the novel global peptide-docking pipeline presented here allows modeling of peptide-protein interactions with much improved accuracy and scope. With further improvements for modeling of increased receptor flexibility and peptide conformational ensemble generation as described above, we should be able to accurately model any interaction that adapts a stable conformation that can be crystallized, as well as explore common features of interactions beyond.

## Materials and methods

### Data set (Table 1 and S1 Table)

Docking performance and analysis was calibrated and assessed on a benchmark of peptide-protein complexes derived from the PeptiDB database [50], filtered according to the following criteria:

1. *Availability of both the complex and the free receptor structure*, solved by X-ray crystallography (resolution of the complex ≤2.0Å).
2. *Absence of crystal contacts that could influence the peptide conformation*. In certain cases this further interaction is of biological relevance, leading to receptor multimerization and clustering (e.g. PeptiDB entries involving some of the SH3 domain-peptide interactions, 2AK5 [55], and 2J6F [56]). Since for these cases, obtaining high-resolution models might be challenging without including the symmetry mate, such examples were removed from the dataset.
3. *Absence of large receptor rearrangement upon peptide binding*. Even though the present implementation of PIPER-FlexPepDock does allow for local conformational changes in the receptor (backbone as well as side chains), accurate modeling of more significant movement of the receptor upon peptide binding (e.g. significant loop movement at the binding interface in PeptiDB entry 1D4T [51]) require the development of algorithms for efficient modeling of more significant receptor flexibility, which is beyond the scope of the present study.
4. *Non-redundant dataset*. The criteria above result in a dataset of 42 complexes (S1C Table) that is very similar to the one used in previous studies by different groups [12, 13, 15, 16]. To ensure that no bias towards a certain peptide-receptor would be introduced, we extracted a domain non-redundant set (defined by CATH classification [36]), resulting in the 27 complexes described in this study in detail (Table 1 and S1B Table).

The dataset was further divided into two subsets, based on available information about a peptide binding motif (defined in this study based on ELM [29], http://elm.eu.org): For the motif set (12 complexes) we modeled only the motif part, since it contributes most to binding, and shorter peptides are easier to model. To enable comparison to performance of other protocols, we subsequently also docked the full peptide. For the non-motif set (15 complexes), the full peptide was docked.

Initial calibration set: For initial calibration, we selected a smaller subset of 9 complexes (S1A Table). The established protocol was then validated on the remaining complexes, to ensure similar performance and thereby prevent overfitting of the modeling protocol.

### The steps of the PIPER-FlexPepDock protocol

In the following we provide specific details of the different steps of the PIPER-FlexPepDock protocol. For runline commands, see the Supplementary S1 Text.

#### (1) Generation of peptide conformations using Rosetta fragment picker and Rosetta fixbb design

The Rosetta fragment picker [22] uses a scoring measure composed of a weighted combination of secondary structure propensity, sequence profile similarity and residue propensities for local regions in the Ramachandran plot [57] to map fragments to *vall*, a database of solved high-resolution monomer protein structures (e.g., *vall*.*jul19*.*2011*, available as part of the Rosetta release). Consequently, the mapped fragments are consistent with the peptide sequence (as defined by a sequence similarity profile generated with PSI-BLAST [58] and secondary structure as predicted using PSIPRED [59]; even though PSIPRED was shown to perform quite well for shorter sequences [11], we use the full protein sequence from which the peptide was derived for PSIPRED and PSIBLAST runs, where available). If the preferred secondary structure is already known (e.g. the alpha helical nuclear receptor box motif) it can be provided instead of PSIPRED predictions. Secondary structural information can also be obtained from experimental techniques such as Circular dichroism (CD) spectroscopy, or approximated by residue Ramachandran local region propensities (derived from statistical analysis of high-resolution protein structure [60]). The coordinates of the top fifty assigned fragments are extracted from the PDB, and side chains of residues not identical to those of the query peptide are modeled using the Rosetta fixbb design algorithm [61]. The whole process results in an ensemble of 50 fragments for the query peptide sequence.

#### (2) Rigid body docking using PIPER

Each of the fifty fragments is globally docked onto the receptor using the PIPER Fast Fourier transform (FFT) docking algorithm, as detailed before [24], decomposing the free receptor into independent binding units (either a single domain or repeated, non-decomposable domains; as in Lavi *et al*. [9]). The calculations are performed for each of 70,000 rotations, and one lowest-energy translation for each rotation is retained. For each fragment docking run the top ranked 250 solutions (total 50x250 = 12500 models) are collected for refinement in the next step (see S3 Fig for a comparison of performance using different numbers of top-ranked solutions).

Selection of final model from a PIPER simulation: In order to compare performance of a protocol involving only the first PIPER rigid body docking step (in Table 1), we selected the final models as reported previously (similar to the PeptiDock implementation [24], but without minimization). In short, the models collected are clustered (with radius of 3.5Å C_α_ RMSD), and cluster density is used for ranking and selection of representatives.

#### (3) The Rosetta FlexPepDock refinement algorithm

The FlexPepDock Refinement protocol refines all of the peptide’s degrees of freedom (*i*.*e*. its rigid body orientation as well as backbone dihedral angles), as well as the receptor side chain conformations. Rosetta FlexPepDock refinement was performed as described previously [10], with slight changes: (1) *Sampling*: In our present implementation, we also allowed the receptor backbone to move during minimization steps, to allow for slight readjustment upon binding (compare e.g. Fig 3B and 3C). (2) *Scoring*: Rosetta energy function Talaris2014 [44] was used. Clustering of models was performed as previously described, using a threshold of 2.0Å [32]. The top-scoring member of each cluster (according to reweighted score) was selected as the representative member, and clusters were ranked based on the reweighted score of the representative members (as in Raveh *et al*. [11]).

### Model evaluation criteria

For each global docking run the 10 top ranking clusters were selected as prediction and evaluated for quality based on ligand RMSD (L-RMSD), calculated between the native and model peptide backbone atoms after optimal superimposition of the receptor, as done in the CAPRI assessment [34, 35]. L-RMSD and other measures, such as Fnat and I-RMSD, were calculated using DockQ [62].

### Rosetta release version

The protocol and tests described in this manuscript follow the FlexPepDock protocol, as implemented within the Rosetta weekly release version 2016.20.58704.

### Simulation running time

The processing time for the different stages of the protocol depends on both the length of receptor and the peptide sequence. For example the global docking the carboxy-terminal tail of the ErbB2 Receptor GLDVPV onto the free ERBIN PDZ domain (103 residues) the generation of 50 fragments takes ~8 CPU minutes over an AMD Sun cluster with 300 cores. For the same complex a single PIPER fragment docking simulation takes ~2 minutes and a single refinement run of the PIPER docked model takes ~1 minutes on the same system architecture (~ 1.5 hours to refine all models).

### Protocol availability

The runline commands are provided in the Supplementary S1 Text. The Rosetta software is available for free to the academic community. The details regarding downloading and installation is available at https://www.rosettacommons.org. PIPER FFT rigid body docking is available as part of the protein-protein docking server ClusPro (PeptiDock at https://peptidock.cluspro.org).

## Supporting information

S1 Fig Global peptide docking energy landscapes for the full dataset (accompanies Fig 3). Top line: PIPER rigid body docking of peptide fragments onto the unbound receptor structure; Middle lines: FlexPepDock refinement of the PIPER docked fragments on the unbound rigid (second line) and flexible (third line) receptor structure; Bottom line: PIPER-FlexPepDock results starting from a bound receptor structure.
(TIFF)

S2 Fig Fragment quality is significantly better for shorter, motif-defined peptide segments (accompanies Fig 2A). Distributions of fragments backbone RMSD values relative to the bound peptide conformations for the motif segments and corresponding full length peptides. The motif set complexes 1JWG and 1TP5 are not added as in these cases the motif covers the whole peptide.
(PDF)

S3 Fig Performance of PIPER-FPD with different number of top PIPER models selected for the refinement stage. Distributions of L-RMSDs of the best models among top 10 ranking clusters for runs using the bound receptor structure (BOUND) and the free receptor structure (UNBOUND & UNBOUND-MIN), the latter including also receptor flexibility in the final refinement step (only the motif region was modeled for the 12 complexes with known motif). The number of PIPER models taken for the FlexPepDock refinement step is shown below each boxplot. Based on these results, we determined a cutoff of 250 models for optimal tradeoff between performance and running time.
(PDF)

S1 Table Details of the datasets of peptide-protein complexes, including modeling results for PIPER-FlexPepDock and other peptide docking protocols (accompanies Table 1). (A) Calibration set (n = 9 complexes); (B) Non-redundant set (n = 27 complexes); (C) Redundant set (n = 42 complexes).
(XLSX)

S2 Table Median fragment-native Backbone-RMSD values for the PeptiDock set complexes obtained using Rosetta fragment picker and the motif-based fragment generation approach used in PeptiDock [24]. (PDF)

S1 Text Runline commands used in this study. (DOCX)

## References

[1] Pawson T, Nash P. Assembly of cell regulatory systems through protein interaction domains. Science. 2003;300(5618):445–52. doi: 10.1126/science.1083653 12702867 10.1126/science.1083653

[2] Petsalaki E, Russell RB. Peptide-mediated interactions in biological systems: new discoveries and applications. Curr Opin Biotechnol. 2008;19(4):344–50. doi: 10.1016/j.copbio.2008.06.004 18602004 10.1016/j.copbio.2008.06.004

[3] Neduva V, Linding R, Su-Angrand I, Stark A, de Masi F, Gibson TJ, et al Systematic discovery of new recognition peptides mediating protein interaction networks. PLoS Biol. 2005;3(12):e405 doi: 10.1371/journal.pbio.0030405 16279839 10.1371/journal.pbio.0030405 PMC1283537

[4] Vacic V, Oldfield CJ, Mohan A, Radivojac P, Cortese MS, Uversky VN, et al Characterization of molecular recognition features, MoRFs, and their binding partners. J Proteome Res. 2007;6(6):2351–66. doi: 10.1021/pr0701411 17488107 10.1021/pr0701411 PMC2570643

[5] Gamble TR, Vajdos FF, Yoo S, Worthylake DK, Houseweart M, Sundquist WI, et al Crystal structure of human cyclophilin A bound to the amino-terminal domain of HIV-1 capsid. Cell. 1996;87(7):1285–94. 8980234 10.1016/s0092-8674(00)81823-1

[6] London N, Raveh B, Schueler-Furman O. Druggable protein-protein interactions—from hot spots to hot segments. Curr Opin Chem Biol. 2013;17(6):952–9. doi: 10.1016/j.cbpa.2013.10.011 24183815 10.1016/j.cbpa.2013.10.011

[7] Trabuco LG, Lise S, Petsalaki E, Russell RB. PepSite: prediction of peptide-binding sites from protein surfaces. Nucleic Acids Res. 2012;40(Web Server issue):W423–7. doi: 10.1093/nar/gks398 22600738 10.1093/nar/gks398 PMC3394340

[8] Saladin A, Rey J, Thevenet P, Zacharias M, Moroy G, Tuffery P. PEP-SiteFinder: a tool for the blind identification of peptide binding sites on protein surfaces. Nucleic Acids Res. 2014;42(Web Server issue):W221–6. doi: 10.1093/nar/gku404 24803671 10.1093/nar/gku404 PMC4086095

[9] Lavi A, Ngan CH, Movshovitz-Attias D, Bohnuud T, Yueh C, Beglov D, et al Detection of peptide-binding sites on protein surfaces: the first step toward the modeling and targeting of peptide-mediated interactions. Proteins. 2013;81(12):2096–105. doi: 10.1002/prot.24422 24123488 10.1002/prot.24422 PMC4183195

[10] Raveh B, London N, Schueler-Furman O. Sub-angstrom modeling of complexes between flexible peptides and globular proteins. Proteins. 2010;78(9):2029–40. doi: 10.1002/prot.22716 20455260 10.1002/prot.22716

[11] Raveh B, London N, Zimmerman L, Schueler-Furman O. Rosetta FlexPepDock ab-initio: simultaneous folding, docking and refinement of peptides onto their receptors. PLoS One. 2011;6(4):e18934 doi: 10.1371/journal.pone.0018934 21572516 10.1371/journal.pone.0018934 PMC3084719

[12] Trellet M, Melquiond AS, Bonvin AM. A unified conformational selection and induced fit approach to protein-peptide docking. PLoS One. 2013;8(3):e58769 doi: 10.1371/journal.pone.0058769 23516555 10.1371/journal.pone.0058769 PMC3596317

[13] Schindler CE, de Vries SJ, Zacharias M. Fully Blind Peptide-Protein Docking with pepATTRACT. Structure. 2015;23(8):1507–15. doi: 10.1016/j.str.2015.05.021 26146186 10.1016/j.str.2015.05.021

[14] Ben-Shimon A, Niv MY. AnchorDock: Blind and Flexible Anchor-Driven Peptide Docking. Structure. 2015;23(5):929–40. doi: 10.1016/j.str.2015.03.010 25914054 10.1016/j.str.2015.03.010

[15] Kurcinski M, Jamroz M, Blaszczyk M, Kolinski A, Kmiecik S. CABS-dock web server for the flexible docking of peptides to proteins without prior knowledge of the binding site. Nucleic Acids Res. 2015;43(W1):W419–24. doi: 10.1093/nar/gkv456 25943545 10.1093/nar/gkv456 PMC4489223

[16] Yan C, Xu X, Zou X. Fully Blind Docking at the Atomic Level for Protein-Peptide Complex Structure Prediction. Structure. 2016;24(10):1842–53. doi: 10.1016/j.str.2016.07.021 27642160 10.1016/j.str.2016.07.021 PMC5080282

[17] Peterson LX, Roy A, Christoffer C, Terashi G, Kihara D. Modeling disordered protein interactions from biophysical principles. PLoS Comput Biol. 2017;13(4):e1005485 doi: 10.1371/journal.pcbi.1005485 28394890 10.1371/journal.pcbi.1005485 PMC5402988

[18] Dominguez C, Boelens R, Bonvin AM. HADDOCK: a protein-protein docking approach based on biochemical or biophysical information. J Am Chem Soc. 2003;125(7):1731–7. doi: 10.1021/ja026939x 12580598 10.1021/ja026939x

[19] de Vries SJ, Rey J, Schindler CEM, Zacharias M, Tuffery P. The pepATTRACT web server for blind, large-scale peptide-protein docking. Nucleic Acids Res. 2017. 10.1093/nar/gkx335 PMC5570166 28460116

[20] Webb B, Sali A. Comparative Protein Structure Modeling Using MODELLER. Curr Protoc Bioinformatics. 2014;47:5 6 1–32. 25199792 10.1002/0471250953.bi0506s47

[21] Trott O, Olson AJ. AutoDock Vina: improving the speed and accuracy of docking with a new scoring function, efficient optimization, and multithreading. J Comput Chem. 2010;31(2):455–61. doi: 10.1002/jcc.21334 19499576 10.1002/jcc.21334 PMC3041641

[22] Gront D, Kulp DW, Vernon RM, Strauss CE, Baker D. Generalized fragment picking in Rosetta: design, protocols and applications. PLoS One. 2011;6(8):e23294 doi: 10.1371/journal.pone.0023294 21887241 10.1371/journal.pone.0023294 PMC3160850

[23] Venkatraman V, Yang YD, Sael L, Kihara D. Protein-protein docking using region-based 3D Zernike descriptors. BMC Bioinformatics. 2009;10:407 doi: 10.1186/1471-2105-10-407 20003235 10.1186/1471-2105-10-407 PMC2800122

[24] Porter KA, Bing X, Beglov D, Bohnuud T, Alam B, Schueler-Furman O, et al ClusPro PeptiDock: Efficient global docking of peptide recognition motifs using FFT. Bioinformatics. 2017;doi: 10.1093/bioinformatics/btx216 28430871 10.1093/bioinformatics/btx216 PMC5860028

[25] Berman HM, Westbrook J, Feng Z, Gilliland G, Bhat TN, Weissig H, et al The Protein Data Bank. Nucleic Acids Res. 2000;28(1):235–42. 10592235 10.1093/nar/28.1.235 PMC102472

[26] Kozakov D, Beglov D, Bohnuud T, Mottarella SE, Xia B, Hall DR, et al How good is automated protein docking? Proteins. 2013;81(12):2159–66. doi: 10.1002/prot.24403 23996272 10.1002/prot.24403 PMC3934018

[27] Brooks BR, Brooks CL 3rd, Mackerell AD Jr., Nilsson L, Petrella RJ, Roux B, et al CHARMM: the biomolecular simulation program. J Comput Chem. 2009;30(10):1545–614. doi: 10.1002/jcc.21287 19444816 10.1002/jcc.21287 PMC2810661

[28] Kozakov D, Brenke R, Comeau SR, Vajda S. PIPER: an FFT-based protein docking program with pairwise potentials. Proteins. 2006;65(2):392–406. doi: 10.1002/prot.21117 16933295 10.1002/prot.21117

[29] Dinkel H, Van Roey K, Michael S, Kumar M, Uyar B, Altenberg B, et al ELM 2016-data update and new functionality of the eukaryotic linear motif resource. Nucleic Acids Res. 2016;44(D1):D294–300. doi: 10.1093/nar/gkv1291 26615199 10.1093/nar/gkv1291 PMC4702912

[30] Puntervoll P, Linding R, Gemund C, Chabanis-Davidson S, Mattingsdal M, Cameron S, et al ELM server: A new resource for investigating short functional sites in modular eukaryotic proteins. Nucleic Acids Res. 2003;31(13):3625–30. 12824381 10.1093/nar/gkg545 PMC168952

[31] Messih MA, Lepore R, Tramontano A. LoopIng: a template-based tool for predicting the structure of protein loops. Bioinformatics. 2015;31(23):3767–72. doi: 10.1093/bioinformatics/btv438 26249814 10.1093/bioinformatics/btv438 PMC4653384

[32] Gray JJ, Moughon S, Wang C, Schueler-Furman O, Kuhlman B, Rohl CA, et al Protein-protein docking with simultaneous optimization of rigid-body displacement and side-chain conformations. J Mol Biol. 2003;331(1):281–99. 12875852 10.1016/s0022-2836(03)00670-3

[33] Lensink MF, Velankar S, Wodak SJ. Modeling protein-protein and protein-peptide complexes: CAPRI 6th edition. Proteins. 2017;85(3):359–77. doi: 10.1002/prot.25215 27865038 10.1002/prot.25215

[34] Mendez R, Leplae R, De Maria L, Wodak SJ. Assessment of blind predictions of protein-protein interactions: current status of docking methods. Proteins. 2003;52(1):51–67. doi: 10.1002/prot.10393 12784368 10.1002/prot.10393

[35] Mendez R, Leplae R, Lensink MF, Wodak SJ. Assessment of CAPRI predictions in rounds 3–5 shows progress in docking procedures. Proteins. 2005;60(2):150–69. doi: 10.1002/prot.20551 15981261 10.1002/prot.20551

[36] Pearl FM, Bennett CF, Bray JE, Harrison AP, Martin N, Shepherd A, et al The CATH database: an extended protein family resource for structural and functional genomics. Nucleic Acids Res. 2003;31(1):452–5. 12520050 10.1093/nar/gkg062 PMC165509

[37] Rohl CA, Strauss CE, Misura KM, Baker D. Protein structure prediction using Rosetta. Methods Enzymol. 2004;383:66–93. doi: 10.1016/S0076-6879(04)83004-0 15063647 10.1016/S0076-6879(04)83004-0

[38] Park H, Lee GR, Heo L, Seok C. Protein loop modeling using a new hybrid energy function and its application to modeling in inaccurate structural environments. PLoS One. 2014;9(11):e113811 doi: 10.1371/journal.pone.0113811 25419655 10.1371/journal.pone.0113811 PMC4242723

[39] Vanhee P, Verschueren E, Baeten L, Stricher F, Serrano L, Rousseau F, et al BriX: a database of protein building blocks for structural analysis, modeling and design. Nucleic Acids Res. 2011;39(Database issue):D435–42. doi: 10.1093/nar/gkq972 20972210 10.1093/nar/gkq972 PMC3013806

[40] Li Y, Suino K, Daugherty J, Xu HE. Structural and biochemical mechanisms for the specificity of hormone binding and coactivator assembly by mineralocorticoid receptor. Mol Cell. 2005;19(3):367–80. doi: 10.1016/j.molcel.2005.06.026 16061183 10.1016/j.molcel.2005.06.026

[41] Guhaniyogi J, Robinson VL, Stock AM. Crystal structures of beryllium fluoride-free and beryllium fluoride-bound CheY in complex with the conserved C-terminal peptide of CheZ reveal dual binding modes specific to CheY conformation. J Mol Biol. 2006;359(3):624–45. doi: 10.1016/j.jmb.2006.03.050 16674976 10.1016/j.jmb.2006.03.050 PMC3666561

[42] Todd B, Moore D, Deivanayagam CC, Lin GD, Chattopadhyay D, Maki M, et al A structural model for the inhibition of calpain by calpastatin: crystal structures of the native domain VI of calpain and its complexes with calpastatin peptide and a small molecule inhibitor. J Mol Biol. 2003;328(1):131–46. 12684003 10.1016/s0022-2836(03)00274-2

[43] Remenyi A, Good MC, Bhattacharyya RP, Lim WA. The role of docking interactions in mediating signaling input, output, and discrimination in the yeast MAPK network. Mol Cell. 2005;20(6):951–62. doi: 10.1016/j.molcel.2005.10.030 16364919 10.1016/j.molcel.2005.10.030

[44] Leaver-Fay A, O'Meara MJ, Tyka M, Jacak R, Song Y, Kellogg EH, et al Scientific benchmarks for guiding macromolecular energy function improvement. Methods Enzymol. 2013;523:109–43. doi: 10.1016/B978-0-12-394292-0.00006-0 23422428 10.1016/B978-0-12-394292-0.00006-0 PMC3724755

[45] Rego N, Koes D. 3Dmol.js: molecular visualization with WebGL. Bioinformatics. 2015;31(8):1322–4. doi: 10.1093/bioinformatics/btu829 25505090 10.1093/bioinformatics/btu829 PMC4393526

[46] Simons KT, Kooperberg C, Huang E, Baker D. Assembly of protein tertiary structures from fragments with similar local sequences using simulated annealing and Bayesian scoring functions. J Mol Biol. 1997;268(1):209–25. doi: 10.1006/jmbi.1997.0959 9149153 10.1006/jmbi.1997.0959

[47] Ho BK, Dill KA. Folding very short peptides using molecular dynamics. PLoS Comput Biol. 2006;2(4):e27 doi: 10.1371/journal.pcbi.0020027 16617376 10.1371/journal.pcbi.0020027 PMC1435986

[48] Vanhee P, Stricher F, Baeten L, Verschueren E, Lenaerts T, Serrano L, et al Protein-peptide interactions adopt the same structural motifs as monomeric protein folds. Structure. 2009;17(8):1128–36. doi: 10.1016/j.str.2009.06.013 19679090 10.1016/j.str.2009.06.013

[49] Kozakov D, Li K, Hall DR, Beglov D, Zheng J, Vakili P, et al Encounter complexes and dimensionality reduction in protein-protein association. Elife. 2014;3:e01370 doi: 10.7554/eLife.01370 24714491 10.7554/eLife.01370 PMC3978769

[50] London N, Movshovitz-Attias D, Schueler-Furman O. The structural basis of peptide-protein binding strategies. Structure. 2010;18(2):188–99. doi: 10.1016/j.str.2009.11.012 20159464 10.1016/j.str.2009.11.012

[51] Poy F, Yaffe MB, Sayos J, Saxena K, Morra M, Sumegi J, et al Crystal structures of the XLP protein SAP reveal a class of SH2 domains with extended, phosphotyrosine-independent sequence recognition. Mol Cell. 1999;4(4):555–61. 10549287 10.1016/s1097-2765(00)80206-3

[52] Davis IW, Arendall WB 3rd, Richardson DC, Richardson JS. The backrub motion: how protein backbone shrugs when a sidechain dances. Structure. 2006;14(2):265–74. doi: 10.1016/j.str.2005.10.007 16472746 10.1016/j.str.2005.10.007

[53] Song Y, DiMaio F, Wang RY, Kim D, Miles C, Brunette T, et al High-resolution comparative modeling with RosettaCM. Structure. 2013;21(10):1735–42. doi: 10.1016/j.str.2013.08.005 24035711 10.1016/j.str.2013.08.005 PMC3811137

[54] Marcu O, Dodson EJ, Alam N, Sperber M, Kozakov D, Lensink MF, et al FlexPepDock lessons from CAPRI peptide-protein rounds and suggested new criteria for assessment of model quality and utility. Proteins. 2017;85(3):445–62. doi: 10.1002/prot.25230 28002624 10.1002/prot.25230 PMC6618814

[55] Jozic D, Cardenes N, Deribe YL, Moncalian G, Hoeller D, Groemping Y, et al Cbl promotes clustering of endocytic adaptor proteins. Nat Struct Mol Biol. 2005;12(11):972–9. doi: 10.1038/nsmb1000 16228008 10.1038/nsmb1000

[56] Moncalian G, Cardenes N, Deribe YL, Spinola-Amilibia M, Dikic I, Bravo J. Atypical polyproline recognition by the CMS N-terminal Src homology 3 domain. J Biol Chem. 2006;281(50):38845–53. doi: 10.1074/jbc.M606411200 17020880 10.1074/jbc.M606411200

[57] Ramachandran GN, Ramakrishnan C, Sasisekharan V. Stereochemistry of polypeptide chain configurations. J Mol Biol. 1963;7:95–9. 13990617 10.1016/s0022-2836(63)80023-6

[58] Altschul SF, Madden TL, Schaffer AA, Zhang J, Zhang Z, Miller W, et al Gapped BLAST and PSI-BLAST: a new generation of protein database search programs. Nucleic Acids Res. 1997;25(17):3389–402. 9254694 10.1093/nar/25.17.3389 PMC146917

[59] Ward JJ, McGuffin LJ, Buxton BF, Jones DT. Secondary structure prediction with support vector machines. Bioinformatics. 2003;19(13):1650–5. 12967961 10.1093/bioinformatics/btg223

[60] Berman HM, Battistuz T, Bhat TN, Bluhm WF, Bourne PE, Burkhardt K, et al The Protein Data Bank. Acta Crystallogr D Biol Crystallogr. 2002;58(Pt 6 No 1):899–907. 12037327 10.1107/s0907444902003451

[61] Kuhlman B, Dantas G, Ireton GC, Varani G, Stoddard BL, Baker D. Design of a novel globular protein fold with atomic-level accuracy. Science. 2003;302(5649):1364–8. doi: 10.1126/science.1089427 14631033 10.1126/science.1089427

[62] Basu S, Wallner B. DockQ: A Quality Measure for Protein-Protein Docking Models. PLoS One. 2016;11(8):e0161879 doi: 10.1371/journal.pone.0161879 27560519 10.1371/journal.pone.0161879 PMC4999177

