# Supplementary material for: High-resolution global peptide-protein docking using fragments-based PIPER-FlexPepDock
Source: PLoS Comput Biol. 2017 Dec 27;13(12):e1005905. doi: 10.1371/journal.pcbi.1005905 (PMC5760072; doi:10.1371/journal.pcbi.1005905)
Supplement: S2 Fig — Distributions of fragments backbone RMSD values relative to the bound peptide conformations for the motif segments and corresponding full length peptides. The motif set complexes 1JWG and 1TP5 are not added as in these cases the motif covers the whole peptide. (PDF) [file pcbi.1005905.s002.pdf]

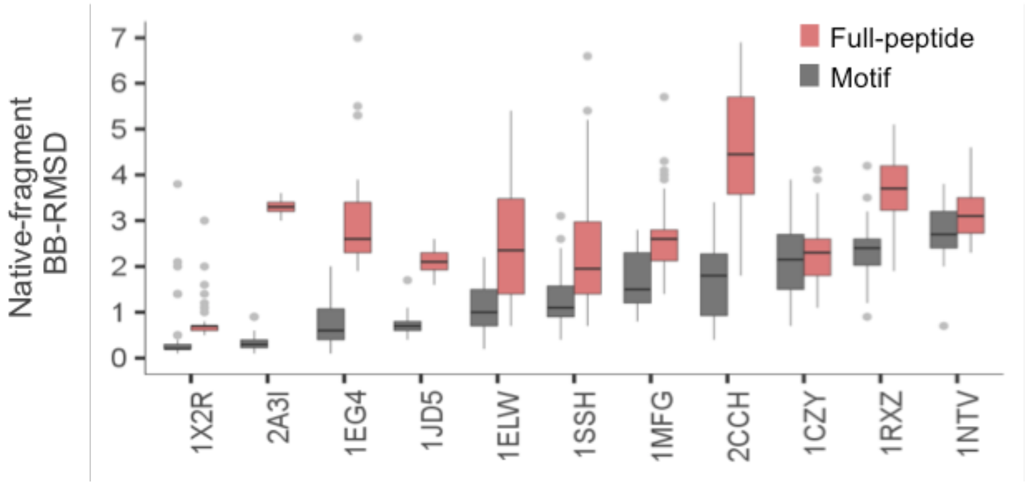

S2 Fig. Fragment quality is significantly better for shorter, motif-defined peptide segments (accompanies **Fig 2A**): Distributions of fragments backbone RMSD values relative to the bound peptide conformations for the motif segments and corresponding full length peptides. The motif set complexes 1JWG and 1TP5 are not added as in these cases the motif covers the whole peptide.
