# Supplementary material for: High-resolution global peptide-protein docking using fragments-based PIPER-FlexPepDock
Source: PLoS Comput Biol. 2017 Dec 27;13(12):e1005905. doi: 10.1371/journal.pcbi.1005905 (PMC5760072; doi:10.1371/journal.pcbi.1005905)
Supplement: S3 Fig — Distributions of L-RMSDs of the best models among top 10 ranking clusters for runs using the bound receptor structure (BOUND) and the free receptor structure (UNBOUND & UNBOUND-MIN), the latter including also receptor flexibility in the final refinement step (only the motif region was modeled for the 12 complexes with known motif). The number of PIPER models taken for the FlexPepDock refinement step is shown below each boxplot. Based on these results, we determined a cutoff of 250 models for optimal tradeoff between performance and running time. (PDF) [file pcbi.1005905.s003.pdf]

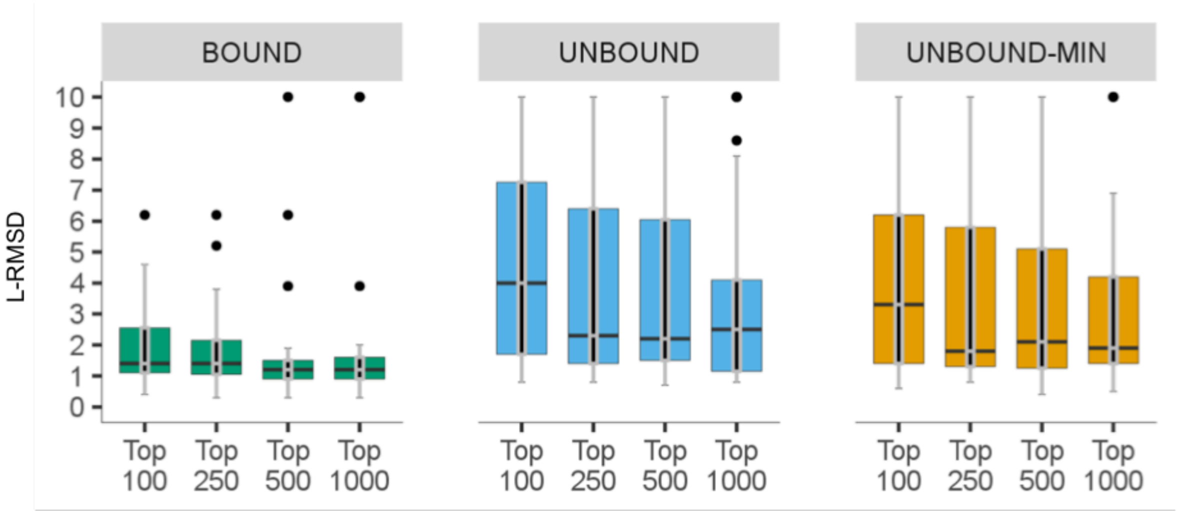

S3 Fig. Performance of the PIPER-FPD with different number of top PIPER models selected for the refinement stage. Distributions of L-RMSDs of the best models among top 10 ranking clusters for runs using the bound receptor structure (BOUND) and the free receptor structure (UNBOUND & UNBOUND-MIN), the latter including also receptor flexibility in the final refinement step (only the motif region was modeled for the 12 complexes with known motif). The number of PIPER models taken for the FlexPepDock refinement step is shown below each boxplot. Based on these results, we determined a cutoff of 250 models for optimal tradeoff between performance and running time.
