# Supplementary material for: High-resolution global peptide-protein docking using fragments-based PIPER-FlexPepDock
Source: PLoS Comput Biol. 2017 Dec 27;13(12):e1005905. doi: 10.1371/journal.pcbi.1005905 (PMC5760072; doi:10.1371/journal.pcbi.1005905)
Supplement: S2 Table — (PDF) [file pcbi.1005905.s005.pdf]

S2 Table. Median fragment-native Backbone-RMSD values for the PeptiDock set complexes obtained using Rosetta fragment picker and the motif-based fragment generation approach (used in PeptiDock (24)).

PDB ID		Peptide sequence (defined by PeptiDock)	Fragment quality [a]	
Complex	Free Receptor		Rosetta fragment picker	PeptiDock sequence – motif based fragment picker
1D4T:A	1D1ZA	TIYAQV	2.4	**1.9**[b]
1SSH:A	1OOTA	PAMPAR	2.0	2.1
1MFG:A	2H3LA	LDVPV	1.4	1.4
2H9M:A	2H14A	ARTKQ	**2.1**	2.4
2FOJ:A	2FAWA	RAHSS	**1.6**	2.0
2HPL:A	2HPJA	DDLYG	**1.7**	2.2
1CZY:A	1CA4A	PQQATDD	2.4	2.5
1JD5:A	1JDA	AIAYF	1.4	1.5
2VJ0:A	1B9KA_1	WVTFE	**1.1**	2.1
2VJ0:A	1B9KA_2	FEDNF	**2.0**	2.5
2C3I:B	2J2IB	RRRHPS	2.5	2.4
2CCH:B	1H1RB	KGRRL	**1.5**	1.9
1EG4:A	1EG3A	RSPPPY	1.6	1.8
1RXZ:A	1RWZA	QATLERWF	2.9	2.8
1ER8:E	4PAEA	HLLVY	1.8	2.0
1JWG:A	1JWFA	DEDLL	2.1	**1.6**

[a] Similarity between fragments and bound peptide conformation: Median backbone RMSD (Å)

[b] In bold: significantly better fragments (Δ≥0.3 Å)
