# Supplementary material for: High-resolution global peptide-protein docking using fragments-based PIPER-FlexPepDock
Source: PLoS Comput Biol. 2017 Dec 27;13(12):e1005905. doi: 10.1371/journal.pcbi.1005905 (PMC5760072; doi:10.1371/journal.pcbi.1005905)
Supplement: S1 Text — (DOCX) [file pcbi.1005905.s006.docx]

**Supplementary Text**

**Runline commands**

The runline commands for the different stages are given below:

1. **Fragment generation using fragment picker:**

The make_fragments.pl script is used to run PSI-BLAST and PSIPRED to generate the peptide secondary structure and the sequence similarity profile:

$make_fragments.pl -verbose peptide.fasta

Rosetta fragment picker is used to assign fragments consistent with the predicted secondary structure and sequence profile to the vall database of high-resolution protein fragments:

$fragment_picker.linuxgccrelease -database rosetta_database -in:file:vall vall.jul19.2011 -in:file:checkpoint pep_seq.checkpoint -frags:frag_sizes 6 -frags:n_candidates 2000 -frags:n_frags 50 -frags:ss_pred pep_seq.psipred_ss2 psipred -frags:scoring:config psi_L1.cfg -frags:bounded_protocol true

These assigned fragments are extracted from the Protein Data Bank (including the side-chains). The non-identical residues are mutated using the Rosetta fixbb design protocol:

$fixbb.linuxgccrelease -database rosetta_database -in:file:s fragment_1.pdb -resfile mutation_resfile -ex1 -ex2 -use_input_sc

**2. PIPER Docking:**

*Step I:* preprocessing the input receptor and fragments using pdbprep.pl and pdbnmd.pl:

$perl pdbprep.pl receptor.pdb
$perl pdbnmd.pl receptor.pdb '?'

Each of the 50 fragments is similarly processed.

*Step II*: Running PIPER FFT docking:

$piper.acpharis.omp.20120803 -vv -c1.0 -k4 --msur_k=1.0 --maskr=1.0 -T FFTW_EXHAUSTIVE -R 70000 -t 1 -p atoms.0.0.4.prm.ms.3cap+0.5ace.Hr0rec -f coeffs.0.0.4.motif -r rot70k.0.0.4.prm receptor_nmin.pdb fragment1_nmin.pdb >piper.log

Each of the fragments is docked onto the receptor.

*Step III*: Top scoring 250 PIPER models are extracted:

$python apply_ftresult.py -i PIPER_model_ID ft.000.00 rot70k.0.0.4.prm fragment1_nmin.pdb --out-prefix PIPER_model_ID

Where PIPER_model_ID is an integer value assigned to each transformation for a fragment.

**3. FlexPepDock Refinement:**

*Step I:* prepacking the PIPER model

In the PIPER docked model the receptor is replaced with a prepacked receptor. A single prepacked receptor is used.

*Step II: Running refinement*

$FlexPepDocking.mpi.linuxgccrelease -database rosetta_database -in:file:s PIPER_model_1.pdb -scorefile score.sc -min_receptor_bb -lowres_preoptimize -pep_refine -flexpep_score_only -ex1 -ex2aro -use_input_sc -unboundrot free_receptor.pdb

Where PIPER_model_1.pdb is the prepacked model.

**4. Clustering:**

The top scoring 1% refined models (125) are clustered using the Rosetta cluster application:

$cluster.linuxgccrelease -in:file:silent decoys.silent top_model_list -in:file:silent_struct_type binary -database $PATH_TO_DB -cluster:radius 2.0 -in:file:fullatom -tags `cat top_refined_list` -silent_read_through_errors

The clusters are ranked based on the top scoring decoys from each clusters, based on reweighted score, and top ranking 10 clusters are selected as putative models.
